# Clinicopathological and prognostic significance of Ki-67, caspase-3 and p53 expression in gastric carcinomas

**DOI:** 10.3892/ol.2013.1532

**Published:** 2013-08-19

**Authors:** LI-JUN XIAO, SHUANG ZHAO, EN-HONG ZHAO, XIN ZHENG, WEN-FENG GOU, YASUO TAKANO, HUA-CHUAN ZHENG

**Affiliations:** 1 Department of Biochemistry and Molecular Biology, College of Basic Medicine, Institute of Pathology and Pathophysiology, China Medical University, Shenyang, Liaoning, P.R. China; 2 Department of Immunology, Chengde Medical College, Chengde, Hebei, P.R. China; 3 Department of Surgical Oncology, The First Affiliated Hospital of Chengde Medical College, Chengde, Hebei, P.R. China; 4 Clinical Cancer Institute, Kanagawa Cancer Center, Yokohama, Kanagawa, Japan

**Keywords:** gastric carcinoma, Ki-67, caspase-3, p53, clinicopathological significance, prognosis

## Abstract

The understanding of proliferative and apoptotic changes has aided the improvement of the diagnosis, treatment and prevention of gastric cancer. The present study aimed to investigate the clinicopathological and prognostic significance of Ki-67, caspase-3 and p53 in gastric cancer. The expression levels of Ki-67, caspase-3 and p53 were evaluated on tissue microarrays of gastric carcinomas specimens by immunohistochemistry and compared with the clinicopathological parameters and survival time of the patients. It was observed that the elder or male patients with gastric cancer showed p53 overexpression compared with the younger or female patients, respectively (P<0.05). The expression of Ki-67 and p53 was positively associated with tumor-node-metastasis (TNM) staging (P<0.05). There was higher caspase-3 and p53 expression in the intestinal-type compared with the diffuse-type of carcinomas (P<0.05). There was a positive correlation among Ki-67, caspase-3 and p53 expression in gastric cancer (P<0.05). A Kaplan-Meier analysis indicated that there was positive correlation between caspase-3 expression and the adverse prognosis of the patients (P>0.05). Cox’s proportional hazards model indicated that the patient age, gender, depth of invasion, lymphatic invasion, lymph node metastasis, TNM staging, Lauren’s classification and caspase-3 expression were independent prognostic factors for gastric carcinomas (P<0.05). The data indicated that the expression of Ki-67, caspase-3 and p53 may be involved in the progression or differentiation of gastric carcinoma. This expression may be employed as an indicator of the pathobiological behavior and prognosis of gastric carcinomas.

## Introduction

Despite a worldwide decline in incidence and mortality in the last 60 years, gastric cancer remains the fourth most common type of cancer and the second most frequent cause of cancer mortality. Gastric cancer continues to be a major health concern due to the slow decrease in incidence in Asia and the high mortality from diagnosed gastric carcinomas in the West, even though advanced diagnostic and operative techniques are widely applied in clinical practice (1,2). Increased understanding of the proliferative and apoptotic changes in gastric cancer, particularly the identification of novel biomarkers for cancer diagnosis and targets for treatment, may result in the improvement of diagnosis, treatment and prevention.

Ki-67 antigen (also known as MKI67) is in the nuclei of cells in the G_1_, S, G_2_ and mitosis phases of the cell cycle and is associated with ribosomal RNA transcription. During interphase, Ki-67 antigen is exclusively detected within the cell nucleus, whereas in mitosis, the majority of the protein relocates to the surface of the chromosomes. Quiescent or resting cells in the G_0_-phase do not express the Ki-67 antigen (3), making the Ki-67 antigen an excellent operational marker for determining the proliferation of a given cell population and the aggressiveness of malignancies (4). In experimental and clinical practice, Ki-67 and MIB-1 monoclonal antibodies are directed against different epitopes of the same proliferation-related antigen; whereas Ki-67 works only on frozen sections, MIB-1 may also be used on fixed sections (5).

The caspase-3 (CASP3) protein is a member of the cysteine-aspartic acid protease (caspase)/interleukin-1β-converting enzyme (ICE) family. CASP3 is activated directly by caspase-8, -9 and -10 in the apoptotic cell by extrinsic (death ligand) and intrinsic (mitochondrial) pathways to initiate apoptosis. CASP3 is synthesized as an inactive 32 kDa proenzyme and processed during apoptosis into its active form, which is composed of two subunits, p17–20 and p10–12. Activated CASP3 is responsible for the cleavage of poly(ADP-ribose) polymerase (PARP), actin and sterol regulatory element binding protein (SREBP), which are associated with apoptosis (6–8).

The p53 tumor suppressor gene is considered to be central in protecting against the development of cancer. The encoded protein is a master switch that coordinates and concentrates a plethora of stress signals, transforming them into a series of responses, including apoptosis or cell cycle arrest, in response to DNA damage, thereby maintaining genetic stability in the organism (9). Therefore, p53 has been described as ‘the guardian of the genome’ (9,10). The p53 pathway is also involved in regulating metastasis-associated genes, including maspin, kai1, integrin, nm23, matrix metalloproteinase (MMP)-2, MMP-13 and the tissue inhibitor of metalloproteinase-3 (TIMP-3) (10–16). Although p53 inactivation in human cancer is a complex process that depends on the tissue type, p53 dysfunction may disorder the biological events of cancer cells, giving rise to their aggressive phenotypes.

Previously, we observed that p53 and Ki-67 were gradually increased from gastrointestinal mucosa to adenocarcinoma through adenoma. Accumulated p53 expression showed a positive association with the depth of invasion, local invasion via vessels and lymph node metastasis of gastrointestinal adenocarcinoma (GIA). Ki-67 expression was positively correlated with local invasion via vessels and negatively correlated with the dedifferentiation and liver metastasis of GIA (17). The present study investigated the clinicopathological and prognostic significance of Ki-67, CASP3 and p53 to clarify their roles in the regulation of the balance between proliferation and apoptosis.

## Materials and methods

### Patients

This retrospective study was performed on curatively-resected gastric carcinoma specimens collected in Toyama University Hospital (Toyama, Japan) between 1993 and 2006. The patients with gastric carcinomas consisted of 130 males and 301 females (age range, 38–88 years; mean age, 66.4 years). Archival materials were obtained from the Department of Pathology. In 165 cases, tumor development was accompanied by lymph node metastasis. None of the patients underwent chemotherapy, radiotherapy and adjuvant treatment prior to surgery. All patients were followed up by consulting their case documents and by telephone.

### Pathology

All tissues were fixed in 10% neutralized formalin, embedded in paraffin and cut into 4-μm sections stained with hematoxylin and eosin (HE) to confirm the histological diagnosis and microscopic characteristics. The staging for each gastric carcinoma was evaluated according to the Union Internationale le Contre Cancer (UICC) system, indicating the extent of tumor spread (17). The histological architecture was defined in terms of Lauren’s classification (18,19). Furthermore, the tumor size, depth of invasion, lymphatic and venous invasion and lymph node metastasis of the tumors were determined.

### Tissue microarray (TMA)

Representative areas of solid tumor were selected for sampling from HE-stained sections of the selected tumor cases, and 2-mm diameter tissue cores per donor block were punched out and transferred to a recipient block, with a maximum of 48 cores, using a Tissue Microarrayer (KIN-1; Azumaya, Tokyo, Japan). Sections (4-μm thick) were consecutively cut from the microarrays and transferred to poly-lysine-coated glass slides. HE staining was performed for the confirmation of the tumor tissue.

### Immunohistochemistry

Serial sections of TMA were deparaffinized with xylene, rehydrated with alcohol and subjected to immunohistochemical staining with intermittent microwave radiation, as previously described (20). Rabbit anti-human CASP3, rabbit anti-human Ki-67 (NovoCastra, Leica Biosystems Newcastle Ltd., Newcastle Upon Tyne, UK) and mouse anti-human p53 (Dako, Carpinteria, CA, USA) antibodies were used at 1:100 dilution to detect the respective proteins, with anti-rabbit or anti-mouse Envison-PO (Dako) as the secondary antibody. Binding was visualized with 3,3′-diaminobenzidine (DAB) and counterstaining with Mayer’s hematoxylin was performed to aid orientation. Omission of the primary antibody was used as a negative control.

### Immunoreactivity for Ki-67, p53 and CASP3

In total, 100 cells from five representative fields of each section were randomly selected and counted in a blinded manner by two independent observers (L. Xiao and H.C. Zheng). Inconsistent data were discussed by the observers until final agreements were reached. The expression positivity was graded and counted as follows: 0, negative; 1, 1–50%; 2, 50–74%; and 3, ≥75%. The staining intensity score was graded as follows: 1, weak; 2, intermediate; and 3, strong. The scores for Ki-67, CASP3 and p53 positivity and staining intensity were multiplied to obtain a final score, which determined their expression as follows: −, 0; +, 1–2; ++, 3–4; and +++, 6–9.

### Statistical analysis

The statistical evaluation was performed using Spearman’s correlation test to analyze rank data. Kaplan-Meier survival plots were generated and comparisons between survival curves were made with the log-rank statistic. Cox’s proportional hazards model was used for the multivariate analysis. SPSS 17.0 software (SPSS Inc., Chicago, IL, USA) was applied to analyze all data and P<0.05 was considered to indicate a statistically significant difference.

## Results

**Figure 1:**
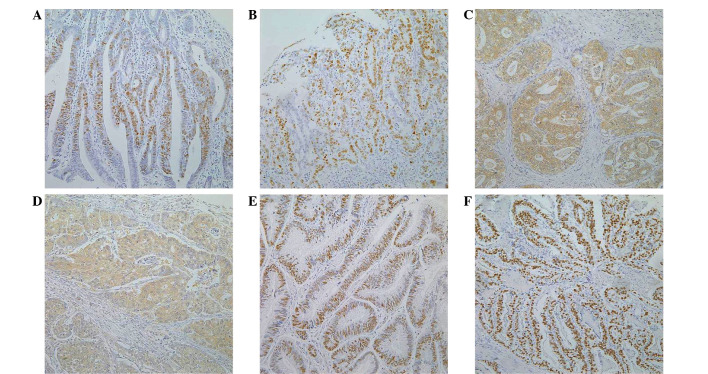
Expression of Ki-67, caspase-3 (CASP3) and p53 in gastric cancer. (A and B) Marked positivity for Ki-67 or (E and F) p53 is localized in the nucleus, while (C and D) CASP3 was observed in the cytoplasm of the gastric cancer cells.

Ki-67 and p53 positivity was clearly localized in the nuclei of the gastric cancer cells, while CASP3 was detected in the cytoplasm of the cancer cells (Fig. 1). Ki-67 expression was positively correlated with tumor-node-metastasis (TNM) staging and p53 expression of gastric cancer (P<0.05), but not with the patients’ age or gender, tumor size, depth of invasion, lymphatic or venous invasion, lymph node metastasis or Lauren’s classification (P>0.05; Table I). CASP3 expression was positively correlated with Ki-67 expression in gastric carcinoma (P<0.05), but not with the patients’ age or gender, tumor size, depth of invasion, lymphatic or venous invasion, lymph node metastasis or TNM staging (P>0.05). There was higher CASP3 expression in the intestinal-type compared with the diffuse-type of carcinoma (P<0.05). The elder or male patients with gastric cancer showed higher p53 expression compared with the younger or female patients, respectively (P<0.05; Table II). p53 expression was positively correlated with TNM staging and the CASP3 expression of gastric cancer (P<0.05), but not with the tumor size, depth of invasion, lymphatic or venous invasion, or lymph node metastasis (P>0.05). The intestinal-type carcinoma samples showed higher p53 expression compared with the diffuse-type samples (P<0.05; Table III).

**Figure 2:**
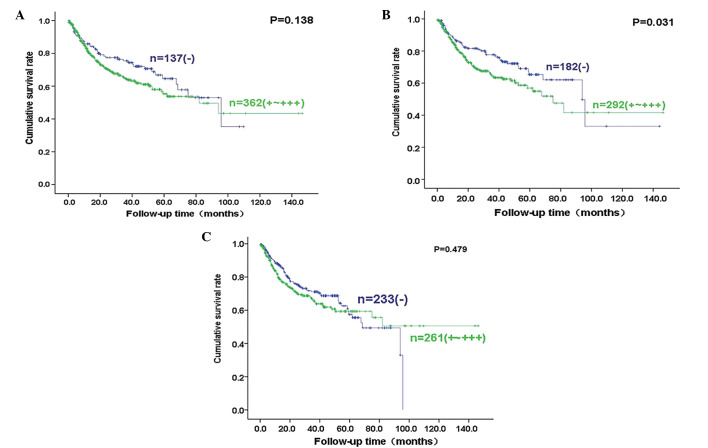
Prognostic significance of Ki-67, caspase-3 (CASP3) and p53 expression in the patients with gastric cancer. Kaplan-Meier curves for the cumulative survival rate of patients with gastric carcinomas according to (A) Ki-67, (B) CASP3 and (C) p53 expression status.

Follow-up information was available for 499 of the gastric carcinoma patients for periods ranging between 0.2 months and 12.2 years (mean, 70.1 months). Fig. 2 shows survival curves stratified according to Ki-67, CASP3 and p53 expression. Univariate analyses using the Kaplan-Meier method indicated that there was a higher cumulative survival rate among carcinoma cases with negative CASP3 expression compared with weak, moderate and strong CASP3 expression (P<0.05), whereas there was no correlation between Ki-67 or p53 expression and the survival rate of the patients with gastric cancer (P>0.05). Cox’s proportional hazard model indicated that the patient age, gender, depth of invasion, lymphatic invasion, lymph node metastasis, TNM staging, Lauren’s classification and CASP3 expression, but not the tumor size, venous invasion and Ki-67 or p53 expression, were independent prognostic factors for gastric carcinomas (P<0.05; Table IV).

## Discussion

Cell proliferative activity is an important factor for assessing the biological behavior of carcinoma, and the identification of proliferating activities in tumors may be useful for predicting clinicopathological and prognostic significance. Ki-67 is a nuclear non-histone protein, which is required for maintaining the cell cycle (22). Our previous study showed gradually increasing expression of Ki-67 from the gastrointestinal mucosa to adenocarcinoma through adenoma (17). In the present study, it was observed that Ki-67 was expressed in 30.4% of gastric cancers and that Ki-67 expression was positively correlated with TNM staging and p53 expression, but not with aggressive parameters such as local invasion, lymph node metastasis and differentiation, which is consistent with previous studies (23–27). Xu *et al*(28) noted that the expression of Ki-67 antigen was significantly associated with distant metastases to the liver, ovary and adrenal gland, but not to the histological type, growth pattern, depth of invasion, histological differentiation or the metastases to local lymph nodes. Several studies have reported that Ki-67 expression was a more valuable independent prognostic predictor for the survival of patients with gastric cancer (24,27,29), which is contrary to the present data.

Normal cells contain only a small amount of caspases in the form of inactive zymogens, and activated caspases have been transformed to proteases via the catalytic activity of enzymes capable of cleaving a number of substrate proteins, resulting in apoptosis. CASP3 is activated by a series of cascade reactions, until eventually DNase (CAD, CPAN or DEF40) is activated, which belongs to the Mg^2+^-dependent endonucleases, and acts as a killer in apoptosis (6,31). Reportedly, CASP3 expression is higher in normal tissues compared with gastric carcinoma tissue (6,32). Hoshi *et al*(32) also observed that the positive rate of CASP3 expression was lower in gastric cancers compared with their adjacent mucosa and gastric adenoma. In the present study, there was higher CASP3 expression in intestinal-type compared with diffuse-type carcinoma, indicating that its aberrant expression underlies the molecular mechanisms of the differentiation of gastric cancer. Additionally, CASP3 was also demonstrated to correlate with the poor prognosis of patients with gastric cancer as an independent factor, in agreement with the study by Isobe *et al*(33). The lack of correlation between CASP3 and aggressive behaviors of gastric cancer was consistent with our previous findings (6). Notably, the positive association between CASP3 and Ki-67 expression suggested the hypothesis that highly proliferating carcinomas may have a high apoptotic potential, contrary to results observed in a previous study (32).

The p53 gene is a tumor suppressor gene located on chromosome 17p13.1 and the single most common target for genetic alterations in human cancer, which is activated in response to genotoxic and non-genotoxic insults to cells (34). Mutated p53 lacks the DNA repair regulation of the cell cycle and results in metabolically stable abnormal protein that accumulates in the nucleus, which may be detected by immunohistochemistry (35). Our previous studies showed that aberrant p53 overexpression was more common in gastric carcinoma than adenoma (17) or intestinal metaplasia (36). In the present study, it was shown that p53 expression was positively correlated with the TNM staging and CASP3 expression of gastric cancer, and that expression was higher in intestinal-type compared with diffuse-type carcinomas, indicating that p53 expression may be involved in the progression and differentiation of gastric cancer. Gonçalves *et al*(35) also noted that p53 expression was more frequent among gastric intestinal-type, differentiated and macroscopically elevated cancers. Significantly shorter survival times were observed in p53-negative patients compared with p53-positive patients. Tzanakis *et al*(37) demonstrated that a more marked expression of p53 was associated with a tumor size of >5 cm, and that advanced stage p53 expression was significantly decreased in poorly-differentiated adenocarcinoma compared with well- or moderately-differentiated adenocarcinoma, which is consistent with the present results. Unlike the present survival data, p53 overexpression was an independent adverse prognostic factor for survival (37).

In summary, the expression of Ki-67, CASP3 and p53 may be involved in the progression or differentiation of gastric carcinoma. These expression levels may be utilized as indicators of the pathobiological behaviors or prognosis of gastric carcinomas.

## Figures and Tables

**Table I:** Correlation between Ki-67 expression and clinicopathological features of gastric carcinomas.

Clinicopathological features	n	Ki-67 expression, n				PR, %	P-value

		−	+	++	+++		
Age, years							0.062
<65	186	60	28	36	62	67.7	
≥65	245	55	29	57	104	77.6	
Gender							0.539
Female	130	38	14	26	52	70.8	
Male	301	77	43	67	114	74.4	
Tumor size, cm							0.146
<4	222	66	25	48	83	70.3	
≥4	209	49	32	45	83	76.6	
Depth of invasion							0.380
T_is-1_	220	57	28	48	87	74.1	
T_2–4_	211	58	29	45	79	72.5	
Lymphatic invasion							0.534
−	271	74	33	60	104	72.7	
+	159	41	24	33	61	74.2	
Venous invasion							0.864
−	373	99	52	86	136	73.5	
+	58	16	5	7	30	72.4	
Lymph node metastasis							0.566
−	262	72	32	62	96	72.5	
+	165	43	25	31	66	73.9	
TNM staging							0.047
0–I	247	59	32	58	98	76.1	
II–IV	184	56	25	35	68	69.6	
Lauren’s classification							0.372
Intestinal-type	214	50	32	46	86	76.6	
Diffuse-type	203	62	23	46	72	69.5	
p53 expression							<0.001
−	179	80	31	36	32	55.3	
+	37	6	9	8	14	83.8	
++	66	7	10	15	34	89.4	
+++	123	11	4	31	77	91.1	

PR, positive rate; T_is_, carcinoma *in situ*; T_1_, lamina propria and submucosa; T_2_, muscularis propria and subserosa; T_3_, exposure to serosa; T_4_, invasion into serosa; TNM, tumor-node-metastasis.

**Table II:** Correlation between nuclear caspase-3 expression and clinicopathological features of gastric carcinomas.

Clinicopathological features	n	CASP3 expression, n				PR, %	P-value

		−	+	++	+++		
Age, years							0.100
<65	173	75	38	32	28	56.6	
≥65	232	81	56	42	53	65.1	
Gender							0.412
Female	125	54	24	22	25	56.8	
Male	280	102	70	52	56	63.6	
Tumor size, cm							0.255
<4	207	88	51	31	37	57.5	
≥4	198	68	43	43	44	65.7	
Depth of invasion							0.141
T_is-1_	205	88	42	31	44	57.1	
T_2–4_	200	68	52	43	37	66.0	
Lymphatic invasion							0.447
−	254	101	51	52	50	60.2	
+	150	55	42	22	31	63.3	
Venous invasion							0.537
−	347	132	81	67	67	62.0	
+	58	24	13	7	14	58.6	
Lymph node metastasis							0.564
−	246	100	55	40	51	59.3	
+	157	56	39	34	28	64.3	
TNM staging							0.919
0–I	232	91	48	37	56	60.8	
II–IV	173	65	46	37	25	62.4	
Lauren’s classification							<0.001
Intestinal-type	201	56	51	36	58	72.1	
Diffuse-type	199	98	43	35	23	50.8	
Ki-67 expression							<0.001
−	103	58	28	14	3	43.7	
+	52	28	8	9	7	46.2	
++	87	29	24	18	16	66.7	
+++	147	34	29	30	54	76.9	

PR, positive rate; T_is_, carcinoma *in situ*; T_1_, lamina propria and submucosa; T_2_, muscularis propria and subserosa; T_3_, exposure to serosa; T_4_, invasion into serosa; TNM, tumor-node-metastasis; CASP3, caspase-3.

**Table III:** Relationship between p53 expression and clinicopathological features of gastric carcinomas.

Clinicopathological features	n	p53 expression, n				PR, %	P-value

		−	+	++	+++		
Age, years							0.032
<65	175	90	16	29	40	48.6	
≥65	247	99	22	38	88	59.9	
Gender							0.027
Female	127	65	12	20	30	48.8	
Male	295	124	26	47	98	58.0	
Tumor size, cm							0.697
<4	214	95	20	35	64	55.6	
≥4	208	94	18	32	64	54.8	
Depth of invasion							0.169
T_is-1_	215	89	20	39	67	58.6	
T_2–4_	207	100	18	28	61	51.7	
Lymphatic invasion							0.861
−	264	119	22	46	77	54.9	
+	157	70	16	20	51	55.4	
Venous invasion							0.721
−	362	160	34	61	107	55.8	
+	60	29	4	6	21	51.7	
Lymph node metastasis							0.532
−	253	113	21	45	74	55.3	
+	164	76	16	21	51	53.7	
TNM staging							0.047
0–I	241	98	22	43	78	59.3	
II–IV	181	91	16	24	50	49.7	
Lauren’s classification							<0.001
Intestinal-type	212	66	22	42	82	68.9	
Diffuse-type	200	119	13	24	44	40.5	
CASP3 expression							0.004
−	142	95	11	12	24	33.1	
+	90	42	4	18	26	53.3	
++	72	25	8	13	26	65.3	
+++	81	12	9	18	42	85.2	

PR, positive rate; T_is_, carcinoma *in situ*; T_1_, lamina propria and submucosa; T_2_, muscularis propria and subserosa; T_3_, exposure to serosa; T_4_, invasion into serosa; TNM, tumor-node-metastasis; CASP3, caspase-3.

**Table IV:** Multivariate analysis of clinicopathological variables for the survival of the patients with gatric carcinomas.

Clinicopathological parameters	Relative risk (95% CI)	P-value
Age (≥65 years)	1.962 (1.342–2.870)	0.001
Gender (female)	1.679 (1.079–2.612)	0.022
Tumor size (>4 cm)	1.511 (0.890–2.566)	0.126
Depth of invasion (T_2–4_)	5.255 (2.376–11.622)	<0.001
Lymphatic invasion (+)	2.193 (1.421–3.383)	<0.001
Venous invasion (+)	1.158 (0.756–1.774)	0.500
Lymph node metastasis (+)	3.629 (1.848–7.126)	<0.001
TNM staging (III–IV)	0.309 (0.138–0.694)	0.004
Lauren’s classification	2.251 (1.457–3.477)	<0.001
Ki-67 expression (+ to +++)	0.982 (0.822–1.172)	0.837
CASP3 expression (+ to +++)	1.277 (1.064–1.533)	0.009
p53 expression (+ to +++)	1.112 (0.947–1.306)	0.194

CI, confidence interval; TNM, tumor-node-metastasis; CASP3, caspase-3.
